# Technology-Assisted Interventions in the Delivery of HIV Prevention, Care, and Treatment Services in Sub-Saharan Africa: Scoping Review

**DOI:** 10.2196/68352

**Published:** 2025-04-15

**Authors:** Louis Henry Kamulegeya, Ivan Kagolo, Brenda Kabakaari, Joan Atuhaire, Racheal Nasamula, J M Bwanika

**Affiliations:** 1 Africa Center for Applied Digital Health Kampala Uganda

**Keywords:** digital health, telehealth, HIV, Sub-Sahara Africa, chatbots, mobile application, mHealth

## Abstract

**Background:**

Sub-Saharan Africa (SSA) accounts for up to 67% of the global HIV burden yet grapples with health system challenges like distant health facilities, low doctor-to-patient ratio, and poor or non-functioning post-hospital follow-up mechanisms. The rising phone ownership and internet penetration in SSA (46% and 67%, respectively) offer an opportunity to leverage technology to address these gaps and drive toward achieving the UNAIDS (Joint United Nations Programme on HIV and AIDS) 95-95-95 targets.

**Objective:**

We undertook a scoping review to understand how digital technologies have been integrated into HIV prevention, care, and treatment services delivery in SSA.

**Methods:**

A scoping review involving 4 databases (PubMed, CINAHL, Cochrane, and Google Scholar) was carried out, encompassing studies related to technology use in the delivery of HIV prevention, care, and treatment published from January 1, 2019, to December 30, 2023. Search terms like “telemedicine,” “telehealth,” “mobile health,” “eHealth,” “mHealth,” “telecommunication,” “mobile application,” and “digital health,” among others, were used. Of the 310 papers identified, 11 were excluded due to duplicity, 299 were from outside SSA and the intervention was not well described, and 149 were due to the year of publication and study type being a literature review or study protocols, leaving 17 papers that were considered for the review.

**Results:**

From the 17 studies summarized, the technologies identified included social media (n=1), interactive voice response (n=1), hotlines (n=1), mobile apps (n=7), health information systems (n=2), chatbots (n=1), and SMS text messages (n=5). Adolescents (11-14 years) and youths (20-35 years) formed the majority of users. The use cases included reminders on facility events, teleconsultations, patient registration, and health information dissemination, among others. Different parameters of individual digital tools were tracked, including feasibility, usability, adoption, and impact on the desired outcome.

**Conclusions:**

The integration of digital technologies in health care can address the known challenges in the delivery of HIV prevention, care, and treatment services, facilitate customization of care to individual needs, and thus increase or diversify options available to patients.

## Introduction

The UNAIDS (Joint United Nations Programme on HIV and AIDS) 2024 global AIDS report showed that Sub-Saharan Africa (SSA) accounted for approximately 67% of the 38.4 million people living with HIV (PLHIV) globally [1]. In response to this burden, governments and development partners have implemented various strategies to increase access to HIV prevention services and provide robust care and treatment. These efforts include initiatives geared toward improving access to HIV testing services like community outreach activities to hotspots and the adoption and roll-out of HIV self-testing, among others [2,3]. Initiatives geared toward increasing access to medical male circumcision and pre-exposure prophylaxis (PrEP) include taking services closer to the people and using influencers or peers, respectively [4,5]. In addition, the expansion of antiretroviral therapy access, viral load monitoring, and screening for opportunistic infections has been prioritized to improve health outcomes for those living with HIV. Despite these efforts and the subsequent gains at the regional level in curbing the HIV/AIDS scourge, SSA remains largely challenged, with a recent UNAIDS report showing only 84% of the target 95% PLHIV being on antiretroviral treatment [6].

Several challenges continue to impede progress toward the UNAIDS 95-95-95 targets in SSA, for example, the low doctor-to-patient ratio, which limits access to credible information for informed decision-making, particularly in rural and underserved areas [7]. The geographic distance to health facilities also poses a significant challenge, often leading to delayed or missed appointments and inadequate follow-up care [8]. Additionally, patients frequently face challenges such as forgetfulness regarding health facility events and a lack of knowledge about the locations of HIV prevention and care services points [9].

Over the last decade, there has been a growing focus on the use of digital technologies in health care, including mobile health (mHealth) apps, telemedicine, and electronic medical records, which have shown promise in improving access to health care and enhancing service delivery efficiency [10,11]. These technologies enable remote monitoring, patient education, and personalized care. Importantly, they allow health systems to reach populations that are often underserved by traditional health care models due to geographic, infrastructural, or socioeconomic barriers. As mobile phone penetration continues to rise in SSA, with over 44% of the population projected to own a smartphone by 2025 [12], the potential for digital health interventions to impact HIV care becomes even more evident.

This nonsystematic review demonstrated how digital technologies have been integrated into HIV prevention, care, and treatment services in SSA. In addition, the review highlights the impact of these digital health interventions in their health domains.

## Methods

### Ethical Considerations

This literature review was conducted in compliance with ethical standards relevant to the analysis of existing data. The study received approval from the Institutional Review Board of the Infectious Diseases Institute, Uganda (IDI-REC-2024-88) and clearance from the Uganda National Council for Science and Technology. Given that this review involved the secondary analysis of publicly available data, it was determined to meet the criteria for nonresearch activities under public health response guidelines. As such, the study did not require the development or registration of a formal research protocol. No new data were collected, and all information analyzed was derived from existing sources, minimizing any potential ethical concerns related to participant confidentiality or data privacy.

### Literature Search and Review

We conducted a scoping review that focused on identifying studies that examined the use of digital health technologies in service delivery models for HIV prevention, care, and treatment services in SSA. In the majority of the use cases, the technologies were used in combination with one another (eg, social media platforms being used for participant recruitment onto a text messaging service for the study). Therefore, the derived themes and categories were not designed to be mutually exclusive but rather to provide the reader with examples of various types of technological approaches incorporated in HIV services delivery in SSA. Searches were performed in 4 databases: PubMed (n=19), Google Scholar (n=262), CINAHL (n=2), and Cochrane Library (n=27). The search strategy combined keywords and MeSH (Medical Subject Headings) terms related to “HIV prevention,” “digital health technologies,” and “sub-Saharan Africa” (Table 1). The search period covered studies published between January 2019 and December 2023. The review excluded gray literature sources as they often lack peer review and a rigorous validation process.

**Table 1:** The search terms and strategy for each database used.

Database	Search terms and strategy
PubMed	(“HIV prevention” AND “digital technologies”) OR (“differentiated service delivery” AND “mHealth”) OR (“telehealth” AND “sub-Saharan Africa”)
Google Scholar	(“HIV prevention” AND “mobile applications”) OR (“digital health” AND “PrEP” AND “Africa”) OR (“eHealth” AND “HIV care”)
CINAHL	(“HIV prevention” AND “digital technologies”) OR (“differentiated service delivery” AND “mHealth”) OR (“telehealth” AND “sub-Saharan Africa”)
Cochrane Library	(“HIV prevention” AND “telemedicine”) OR (“digital innovations” AND “viral suppression”) OR (“mobile health” AND “community-based service delivery”)

### Study Selection

Studies included in the review met the following criteria (Textbox 1).

Studies excluded from the review included those with the following criteria (Textbox 2).

**Inclusion criteria.**

- Published papers in peer-reviewed journals that focused on digital health technologies used as direct service delivery mechanisms for HIV prevention, care, and treatment.
- Studies and reports published between 2019 and 2023.
- Studies conducted in Sub-Saharan Africa.
- Publications in the English language.

**Exclusion criteria.**

- Did not focus on digital health technologies.
- Were not related to HIV service delivery (prevention, care, and treatment).
- Were conducted outside Sub-Saharan Africa.
- Were published before 2019 or after 2023.
- Were not published in English.

A total of 310 studies were identified in the initial database search for which after screening, only 17 studies met the inclusion criteria for our review.

### Data Abstraction

Data abstraction was conducted using an Excel spreadsheet (Microsoft Corp) to capture key study characteristics, including author, year of publication, study type, study participants, and outcomes. To ensure rigor and minimize bias, 2 independent reviewers extracted the data from the selected studies. Any discrepancies in the data abstraction process were resolved by a third reviewer, who acted as a mediator. The third reviewer facilitated discussions to reconcile conflicting data points and ensure consistency in the final dataset.

The extracted data were grouped into categories based on the type of digital technology used (ie, social media, mobile phone apps, health information systems, mobile SMS or SMS text messaging, chatbots, interactive voice response (IVR), and hotline.

## Results

**Figure 1:**
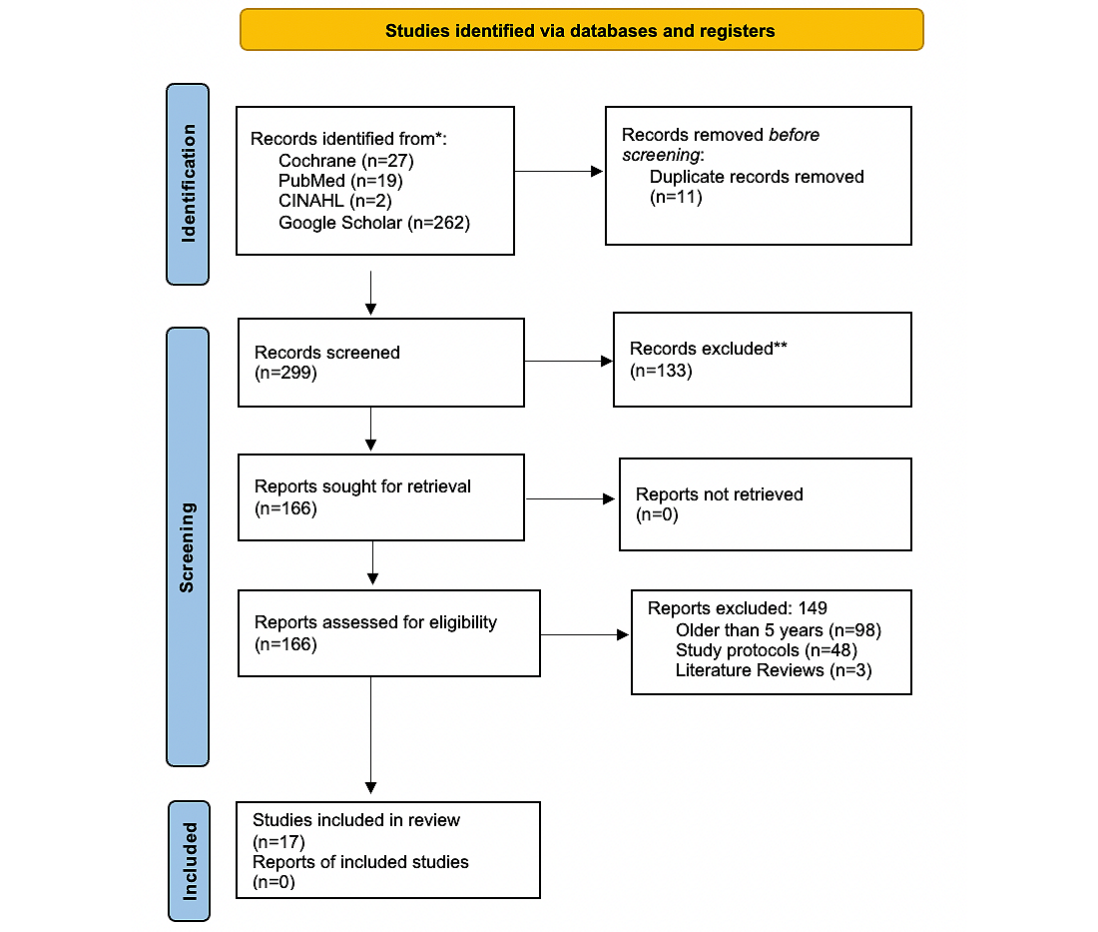
PRISMA (Preferred Reporting Items for Systematic Reviews and Meta-Analyses) flow diagram summarizing the selection process for studies included in the scoping review.

This scoping review was conducted in accordance with the PRISMA-ScR (Preferred Reporting Items for Systematic Reviews and Meta-Analyses extension for Scoping Reviews) guidelines (see Figure 1 for the PRISMA-ScR flow diagram and Multimedia Appendix 1 for the PRISMA-ScR checklist).

### Social Media

Social media included the use of digital platforms to support virtual networking and the sharing of content amongst people [13]. The review showed that social media platforms are pivotal in reaching younger audiences like adolescents and have the potential to reach very large numbers. This is evident in the study done in South Africa among adolescent girls and young women that used social media platforms reaching over 34 million users on awareness and demand generation for oral PrEP [14]. Close group social media channels like Grindr, Facebook, and WhatsApp offer a safe space for sub-populations (eg, men who have sex with men (MSM), female sex workers (FSW), injectable drug users, etc) that often face high levels of stigma, discrimination, and criminalization. This was evident in a non–randomized controlled trial that used social media and peer navigation to promote HIV testing and linkage to care among high-risk young MSM [15]. The study noted an increased uptake of HIV testing with the associated increase in positive yield among the MSM, and also linkage to care was high at 86.1%. One common feature among these 2 studies that used social media is that they used already widely known and commonplace platforms without developing or marketing anything new to the communities. This may explain the easy adoption and usage since these platforms already have subscribers. This is cost-effective (requires less time and money) and will achieve widespread acceptance and use.

### Mobile Apps

Mobile phone apps have been piloted and used in a number of use cases, including promotion of treatment adherence, empowering self-monitoring among patients or users, risk self-assessment for HIV, and gamification apps for information dissemination geared toward behavioral change. A mobile app piloted among MSM and FSW in Dar es Salaam, Tanzania, to increase adherence to PrEP showed that 52% of the app users adhered to the daily PrEP medicines [16]. The use and satisfaction with the app were high, especially for educational content and interactive channels with peer educators. In addition, sub-optimal use of mobile apps among the target users (MSM and FSW) was associated with increased retention (37.7%) of PrEP care [17]. A study that assessed the use of a mobile app for HIV risk assessment among sexually active adults in South Africa showed that keeping the screening and self-assessment questions short and the use of formal language increases the use of such tools [18]. However, a constant theme on data protection and privacy of users comes out strongly with suggestions on password protection of these apps. The mobile app offers the opportunity to reach and engage subpopulations (men and youths) that are often excluded from the traditional health care delivery model owing to the limitations of contact time and space. Digital tools like HIV risk self-assessment and gamification apps create a feeling of self-awareness and empower one to take an informed decision based on long-term health benefits not only to themselves but also to the family [19,20]. Mobile apps can be tailored to be more engaging with gamification. A study by Sabben et al [20] assessed the feasibility and usability of a gamification app among adolescents and found that 90% found the game “very fun” and 97% would recommend it to friends. The adolescents played on average 27 hours; 87% played daily; and 77% played for more than an hour per session. All participants (100%) indicated that they had learned “a lot” and found the information useful, 93% felt more prepared to handle difficult situations, and 97% felt more confident in saying no when pressured to engage in unsafe sex [20]. These findings further solidify the fact that high-tech platforms are highly suited for younger demographics.

### Health Information System and Electronic Medical Records System

Electronic information systems for health are designed to collect data that helps to support planning, management, and decision-making in health facilities and organizations. Electronic health records enable health workers to anticipate client traffic, control workflow, and plan for supplies accordingly through their ability to give reminders and prompts. A study in Malawi showed how electronic health record led to the improvement in retention of HIV-positive clients and viral suppression monitoring through automated alerts and reminders for antiretroviral therapy adherence [21]. Electronic information systems enable connectivity between health facilities, an important aspect in preventing duplicity of patients records due to their mobile nature, and repeated registration on seeking care in a new geographical location is commonplace.

### Interactive Voice Response

IVR involves disseminating pre-recorded voice messages to beneficiaries via mobile phone technology. This may involve using an influencer figure, especially where messages are to a specific demographic, in order to resonate with the audience. The provision for voice listening-in means that the literacy skill of the participant is not a limiting factor. A study in Uganda deployed the IVR tool among adult PLHIV to increase retention in care and clinic appointment adherence and improve viral load suppression, showing 346,286 outbound IVR calls were made to the study participants, with over half (52.8%) listening in to the recording, an indicator of adoption and acceptability [22]. The same study noted that increased use of the IVR was associated with improved performance on a number of indicators tracking the quality of life of the study participants, for example, on mental health domain scores (*P*=.008), and viral suppression (*P*=.006), suggesting that the tool had a positive impact on those who used it regularly. Additionally, these participants also showed better adherence to clinic appointments.

### Mobile SMS

Mobile messages are one of the earliest digital technologies to be incorporated in the routine delivery of health services in SSA. A number of use cases of SMS interventions in the realm of HIV prevention, care, and management exist, as profiled in Multimedia Appendix 2. SMS text messages are a convenient way to disseminate health messages that do not require contact time or space with the target audience. Horvath et al [3] showed that text messaging cascading motivational and behavioral information on HIV self-testing to sexually active males showed a 94% satisfaction rate with up to 86% of participants that received the messages ordering HIV self-testing kits for themselves or partners [3]. SMS text messages have been deployed as nudges to remind beneficiaries of important health facility events like clinic appointments, medicine refills, and pill-taking among others. A randomized control trial by Ybarra et al [23] in Uganda showed that targeting youths with reminder messages on condom use and HIV testing increased both significantly, ie, condom use in the intervention group (adjusted incidence rate ratio 1.68, *P*<.001) and a higher odds of HIV testing in the intervention group: (adjusted odds ratio 2.41, *P*=.03). Another study in Uganda showed an increased uptake in HIV (from 57% to 82%) and syphilis (from 35% to 81%) testing services among female sex workers targeted with reminder messages [24]. Also, the review showed that SMS text messages are pivotal in patient registration and teleconsultation, where provisions for 2-way interactions are provided [21,23].

### Chatbots

A chatbot uses natural language processing to allow for real-time interactions with users on predefined topics. A cross-sectional study by Ntinga et al [25] assessed the feasibility and acceptability of a chatbot among rural adults in driving demand and uptake of HIV testing services. The chatbot was deployed via WhatsApp, WeChat, and Telegram and guided users through their journey of HIV self-testing by offering pretesting counseling, information on how to use HIV self-testing kits, and linkages to service points to access the service. The study findings indicated that users had a positive experience, with 79.2% rating the experience better than with a human counselor and up to 77.5% feeling the conversation was similar to talking to a real person [25]. Privacy, nonjudgmental interactions, and convenience of the chatbot compared to in-person methods were cited as benefits. The study also noted that 17.5% of users that got tested positive through the chatbot were linked to care; 82.8% of those who tested negative wanted to know more about PrEP, which shows the power of self-drive automated platform institutes among people. Outside the realm of research, at a commercial level, chatbots do exist offering self-triage. Symptom checker and advice with examples like Ada and Babyl [26,27]. At the program level, chatbots have been deployed in United States Agency for International Development-funded activities in Uganda to increase reach and engagement through diversifying channels of communication. Notably, the “real kool” campaign was promoting awareness about one’s HIV status and taking on HIV prevention services like condoms and HIV self-testing, among others. The campaign leveraged a chatbot deployed via the WhatsApp platform that offered real-time access to health information, risk self-assessment for HIV, and a directory to HIV self-test kit distribution points [28].

### Hotlines

Hotlines manned by health care professionals (counselors, medical doctors, community health extension workers, etc) have been used in HIV service delivery in SSA countries. These hotlines are used in the remote resolution of clients’ inquiries, virtual follow-ups, and linkages to health services, among others [29]. Horvath et al [3] used a toll-free hotline manned by health workers to offer remote guidance on how to use HIV self-testing kits and facilitate result interpretation. The level of privacy and anonymity hotlines provide allows easy interactions on topics that would otherwise be stigmatizing in physical interaction with a health care provider at a health facility.

## Discussion

### Principal Results

Our scoping review found few studies in SSA where technology-assisted interventions were used for HIV prevention and care. The strategies in these studies included mobile apps, electronic information management systems, mobile SMS, social media, chatbots, gamification apps, interactive voice recording, and voice call-in lines. The target groups the studies covered included FSW, MSM, health care workers, adolescent girls and young women, PLHIV, and youths. The studies showed a high adoption and usage rate for digital technologies and also showed a positive trend toward the desired health outcomes among the study population.

The majority of the studies used a combination of technologies and approaches in the delivery of HIV prevention, care, and treatment services. For example, Hovarth et al [3] used a combination of a voice call-in line for teleconsultations and follow-ups alongside SMS text messages for motivational-behavioral messaging for HIV self-testing demand creation and use. Dietrich [18] leveraged community outreach to recruit study participants onto the mobile apps that provided sexual behavior self-risk assessments. This observation speaks to the fact that digital health innovations are not a stand-alone approach but rather adjuncts to other approaches and interventions aiming at amplifying the outcomes.

It is important to note that for all the profiled 17 studies in the review, the target audiences or beneficiaries were relatively young, ie, adolescents (11-14 years) and young adults (20-35 years). This may speak to the fact that being tech-savvy, this demographic is quick to learn and adopt technological trends compared to older persons who may be resistant to change and often lack digital literacy [24]. This finding could inform behavioral change communicators on the potential digital channels have in reaching this usually left-out audience, given the traditional print and media channels used to cascade health information to the masses [30].

The high adoption and usage rates of these digital technologies in SSA are an indicator of the high access and penetration of mobile phones, which currently stands at 46% according to the Global Health Security Agenda report of 2024 [31]. However, almost all studies pointed out challenges to do with poor network connectivity, especially in rural areas, and the cost of internet bundles as being key barriers to leveraging mobile phone technologies in the delivery of health services [14,18,32,33]. Therefore, this calls for a concerted effort from all stakeholders, including the private sector, like telecommunication companies that could partner with governments to make internet connectivity more accessible, especially for health purposes.

Digital technologies offer beneficiaries the advantages of privacy, confidentiality, and anonymity that allow users to freely express themselves and overcome the stigma commonly experienced within certain subpopulations due to factors like the legal or regulatory environment [16,25]. This allows nondiscriminatory access to health care as a right for any individual irrespective of any socioeconomic background. However, this builds a critical challenge on ensuring secure data privacy for all information electronically collected and stored as part of patient care. A number of SSA countries have established laws and regulations on data protection and privacy to which end-innovators and partners should ensure to align and adhere to during the development and use of digital innovations in health care delivery. The lack of technology data among the non-youth cohort for this study shows that there is an opportunity for deliberate engagement with this group. HIV has become a long-term, manageable health condition due to treatment advances. With technological advances, the number of PLHIV will increase. Therefore, emphasis on how to effectively use technology to engage the older generation in obtaining access to HIV care is needed.

Digital platforms are very flexible and agile and can easily be scaled up with minimal human resource additions, a key aspect of cost-effectiveness during the scale-up phase. Take an example of an interactive HIV risk self-assessment chatbot that can reach over millions of users in one go with just a simple mobile app compared to a single health worker seated at a clinic and can only be able to screen a given number of patients in a queue. The ability to translate content disseminated through digital channels to preferred local languages and that users can interact with the digital tools as and when they need them without the challenges of working hours speaks to the flexibility of these tools. One common feature among some of the studies is the use of already existing digital platforms as delivery channels; for example, Ntinga et al [25] leveraged social media platforms like WhatsApp and Grindr to deploy the chatbot. This facilitates the adoption and ease of use of the innovations, as there is little or no training and onboarding required since they use already widely known and commonplace platforms without developing or marketing anything new.

### Limitations

Due to financial access limitations, some databases were not covered by the scoping review we undertook. Also, our review excluded gray (unpublished) literature, which at times holds information on the application of digital technologies in the delivery of HIV prevention, care, and treatment services in the form of program reports, a domain commonly led through developmental partner projects in SSA.

### Conclusions

Our scoping review showed that the integration of digital technologies in the delivery of HIV prevention, care, and treatment services in SSA is feasible, given the high usage rates. These technologies offer the opportunity to engage audiences typically unserved by the traditional health care model, notably the youth and key populations. The persistent barriers to quality health care in SSA owing to the low doctor-patient ratio and poor or nonfunctional post–health facility follow-up system can be addressed by novel technologies like self-help chatbots and artificial intelligence. Our review demonstrated that technologies have been deployed to serve a plethora of services in HIV prevention, care, and treatment programs, including health information dissemination, nudges in the form of reminders on health facility events, and decision support tools, among others. Therefore, digital tools can be looked at as tools that can be used to increase reach, scalability, and efficiency when delivering HIV prevention, care, and treatment services.

## Appendix

Multimedia Appendix 1 PRISMA-ScR checklist.

Multimedia Appendix 2 Characteristics of studies included in the final review.

## Abbreviations

FSW: female sex workers
IVR: interactive voice response
MeSH: Medical Subject Headings
mHealth: mobile health
MSM: men who have sex with men
PLHIV: people living with HIV
PrEP: pre-exposure prophylaxis
PRISMA-ScR: Preferred Reporting Items for Systematic Reviews and Meta-Analyses extension for Scoping Reviews
SSA: Sub-Saharan Africa
UNAIDS: Joint United Nations Programme on HIV and AIDS
