# Supplementary material for: Technology-Assisted Interventions in the Delivery of HIV Prevention, Care, and Treatment Services in Sub-Saharan Africa: Scoping Review
Source: J Med Internet Res. 2025 Apr 15;27:e68352. doi: 10.2196/68352 (PMC12041816; doi:10.2196/68352)
Supplement: Multimedia Appendix 2 [file jmir_v27i1e68352_app2.docx]

Multimedia Appendix 2

Characteristics of studies included in the final review.

| Author, publication year | Study location, study design, study population, sample size | Digital tools and features | Study measures | Results | Facilitators | Barriers |
| --- | --- | --- | --- | --- | --- | --- |
| Mauka, W, 2020 | Tanzania,  Longitudinal study  MSM, FSW  Median age 27 yrs  Level of education: Primary  20 (FSW: 10, MSM: 10) | Smartphone-based mobile app  Features: educational messages, PrEP adherence reminders, gamification, real-time teleconsultation | Acceptability and usability of the mobile app for PrEP uptake | Half used the app to register their daily pill-taking  On average, one app feature was used daily  74% App usage for teleconsultations  Willingness to recommend the app to peers | Anonymity and safety  Culturally and legally acceptable content on the app | Stigma and discrimination  Internet connectivity  Airtime shortage |
| Thomas, D, 2020 | Tanzania  Longitudinal study  PMTCT health workers  27 (11 from dispensaries, 9 from health centers, 7 from hospitals) | Health information system  Features:  patient registration, SMS reminders, educational content, decision-making support | Acceptability (including attitude and self-efficacy) and impact knowledge of clinical care | Improvement in system attitude and overall acceptability  Health workers in more remote areas (>20 km from the hospital) showed greater improvement in knowledge | Educational messages and prompts embedded within the system  Health workers had a high interest in participating in using the tool and offering feedback | Initial lower levels of acceptability among HWs in hospitals and health centers compared to those in dispensaries |
| Swai, U, 2023 | Tanzania  Mixed methods (quantitative and qualitative data collection)  Children (0-14 years) and adolescents (15-19 years) living with HIV  426 (284 children and 142 adolescents) | Mobile SMS  Features:  Pillbox registers  SMS reminders  Adherence reports to users | Acceptability | 85% of participants wanted SMS reminders  90% of participants positively evaluated the adherence feedback reports | Confidential SMS content helped preserve privacy | Poor network affected SMS delivery  Missed notification due to phone being in silent mode or malfunctioning  Lack of access to electricity  Phone-sharing and lack of privacy, particularly among school-going participants |
| Dietrich, J, 2021 | South Africa  Qualitative research using focus group discussions  Sexually active adults (18-39 years)  Median age: 30 (IQR: 24–34)  75.9% completed high school  29 Participants (men: 14; women: 15) | Smartphone-based mobile application  Features:  Sex-life risk assessment  Reminders | Usability | 93.1% (n=27) downloaded the app  Preferred frequency for surveys was weekly (on weekdays)  Majority preferred formal language for clarity | Password protection for privacy  Incentives (airtime per survey)  Simplicity and ease of use of the app  Personalized feedback encouraged participants to engage | Data costs for internet use  User fatigue with frequent survey requests  Privacy concerns about using SMS or social media for assessments  Survey completion could be hindered by busy schedules and internet unreliability |
| Haberer, J, 2022 | Uganda  Longitudinal study  Adult PLHIV on antiretroviral therapy (ART)  Median age: 34 years  60% had primary education  59% were married  90% were employed  50% of participants had been on ART for more than 6 months  51 (male (n=29, 57%; female (n=22, 43%) | Smartphone-based mobile application  Features:  Daily pill monitor  SMS reminders to clients and social supporters | Feasibility, acceptability, and user experience of the app  Adherence to ART | 94% overall average adherence  92% of clients found SMS reminders very useful  Reduction in clinic time through focused counseling (clinic visits with intervention lasted 4 minutes on average) | High perceived usefulness of the intervention | The cost of implementation was a concern for scale-up  Technical challenges (network delays, SMS errors, and scheduling)  Training needs for users |
| Garofalo,R, 2022 | Nigeria  Nonrandomized controlled trial  High-risk young men (MSM), 15-24 yrs  339 MSM | Social media platforms (WhatsApp, Facebook, and Grindr) | HIV testing uptake and linkage to care | HIV testing increased by 31%–42% in two 6-month periods  Seroprevalence was 10.6% (36 participants tested HIV positive)  86.1% of participants who tested positive were linked to care | Use of commonly used social media platforms for outreach  Peer navigation increased engagement and trust | Legal and regulatory environment (stigma and criminalization of MSM in Nigeria, limiting access to facility-based HIV services)  Concerns about the sustainability and scalability of the intervention, particularly in areas without strong NGO support |
| Briedenhann, E, 2023 | South Africa  Observational study  Adolescent girls and young women (AGYW) aged 15-24 years at risk of HIV infection  42,447 AGYW | Web-based application  Features:  Chatbot  Clinic finder tool: Calculates the nearest PrEP-providing health care facilities  Pill-taking reminders, quizzes, motivational messages, and gamification to support PrEP adherence | Reach: number of individuals reached  Effectiveness: number of AGYW presenting for sexual and reproductive health (SRH) services and PrEP initiation  Adoption: number of health care facilities and mobile clinics implementing the model  Implementation: training provided, materials produced, and interventions carried out  Maintenance: integration of tools and materials into the National Department of Health Resources | Overall reach: over 34 million connections made through multiple communication channels  Effectiveness: of the 16,823 AGYW who presented for SRH services, 14,637 (87%) were initiated on PrEP  Adoption: implemented in 8 health care facilities and 4 mobile clinics across 3 provinces  Implementation: 26 training sessions provided to project staff; youth advisory groups formed  Maintenance: National Department of Health adopted several tools and materials from the project | High engagement via a combination of digital and face-to-face strategies  Community buy-in through stakeholder engagement and youth-centered approaches  Use of peer navigators to assist AGYWs in navigating health care services  Flexibility in adapting to local contexts (cultural, religious, socioeconomic) | Stigma surrounding HIV and sexual health  Long waiting times at health care facilities  Variable levels of internet access among AGYW |
| Feldacker, C, 2022 | South Africa  Prospective, unblinded, noninferiority, randomized, controlled trial  Adult males who underwent VMMC and owned a cell phone  Median age 31 years (IQR 24-37)  Near equal representation from urban (50.1%) and rural (49.9%) areas  1084 males (547 in the 2-way texting (2 wT) arm, 537 in the control arm) | Mobile SMS (2wT)  Features:  Teleconsultation via text  Adverse event monitoring and follow-up | Safety (cumulative adverse events (AEs) ≤ day 14) and workload (number of in-person follow-up visits)  AE rates and SMS response rates | Safety: cumulative AEs identified in 2.3% of 2wT participants vs 1% in control; 2wT demonstrated noninferiority for AE identification (*P*=.13)  Workload reduction: 2wT reduced follow-up visits by 84.8% compared to control (mean visits: 0.22 in 2wT vs 1.34 in control; *P*<.001)  Response rates: daily response rates ranged from 86% on day 3 to 74% on day 13; 94% of 2wT participants responded to at least one SMS | Reduced workload for health care workers by minimizing unnecessary follow-up visits  High participant engagement with SMS responses and the 2wT system  Earlier AE identification due to frequent communication via SMS  Positive patient-provider communication through SMS | Digital literacy among health care workers was low, which could hinder scale-up  Potential patient skepticism about the effectiveness of telehealth |
| Feldacker, C, 2023 | Malawi  Longitudinal study  Clients on ART from urban health centers providing ART services  37,400 stable ART clients | Mobile electronic medical record system (EMRS) app  Features:  Patient registration and barcode scanning for client identification  Task management,  Decision support,  Embedded alerts for HIV care adherence | Adoption, functionality, and usability | Successfully designed and piloted the app with expected improvements in client care and monitoring  Improved integration of ART guidelines into community-based settings  Enhanced client retention and viral suppression monitoring through automated alerts and reminders for ART adherence | High digital literacy among health care workers  Iterative human-centered design process, with participatory feedback from health care providers and M&E staff  Offline capabilities allowing the app to function in low-connectivity areas | Need for integration between the app and static clinic EMRS to ensure timely care alerts  Continued reliance on physical connection to clinic intranet for data synchronization  Challenges with app functionalities like speed |
| Ntinga, X, 2022 | South Africa  A cross-sectional study  Adults (18-47 years), residents in rural communities with high HIV prevalence  120 (Male 61, 50.8%; Female 59, 49.2%)  Education: secondary or high school (67.5%), tertiary institution (27.5%), primary school (1.7%), socioeconomic status: poor (40%), middle (40.8%), rich (19.2%) | Chatbot  Features:  Multiple personalities (2 aged 18-25 years, 2 aged 35-50 years)  User guide on HIVST and results interpretation  Links to additional PrEP information | Feasibility and acceptability of the bot  User interaction with the chatbot, ability to follow instructions for HIVST, chatbot’s capability in guiding self-testing and interpreting results | User experience: 79.2% rated the experience better than with a human counselor, 77.5% felt the conversation was similar to talking to a real person  Linkage to care: 17.5% tested positive and were linked to care; 82.8% of those who tested negative wanted to know more about PrEP | Privacy and non-judgmental interaction (cited as benefits over human counselors)  Ease of use, speed, and functionality in providing guidance and information | Some users felt the chatbot lacked empathy and preferred human interaction in the event of a positive result  Chatbot conversations were unidirectional, limiting the ability to ask questions or engage in deeper conversation  A small portion of users (12.5%) found the responses too fast, making the chatbot feel less human-like |
| Mathenjwa, T, 2020 | South Africa  Mixed methods  Rural, predominantly male, who had tested HIV positive but had not linked to care within a month  Age range: 34-66 years  57% were unemployed  Phase 1: 11 men for focus groups  Phase 2: 12 men for usability testing and 7 men for iterative app design testing  Phase 3: 14 men completed satisfaction surveys and 14 completed in-depth interviews | Smartphone-based mobile app  Features:  Decision support  Story or experience sharing  Counselling | Acceptability, user satisfaction, and linkage to HIV care | User satisfaction: 86% (12/14) felt empowered to make informed health decisions  79% (11/14) rated the app as user-friendly, and 93% (13/14) agreed it appealed to their conscience to seek HIV care  Linkage to care: 11 out of 14 interviewed men linked to HIV care after using the app | Local narratives from men sharing their HIV care journeys helped to personalize and resonate with users  Simple user interface  The app provided autonomy in decision-making, leading to improved user engagement  Referral letters and support from research nurses encouraged linkage to care | Stigma and fear of being seen at the facility  Initial usability challenges with complex app features or content |
| Ybarra, M, 2021 | Uganda  Randomized controlled trial  Youths (18–22 years)  202 participants (54% male) | Mobile SMS  Features:  Daily SMS text messages (5-10 per day for 7 weeks, plus a 1-week booster after 12 weeks)  Interactive elements (questions, badges)  Peer system for discussing content | Feasibility (retention rate)  and acceptability (ratings for content and features)  Behavior changes outcomes (condom use, HIV testing, abstinence) | Messages read by participants averaged 86%  Condom use: significantly higher in the intervention group (aIRR=1.68, *P*<.001)  HIV testing: higher odds in the intervention group (aOR=2.41, *P*=.03)  Abstinence: no significant difference between the intervention and control groups (aOR=1.08, *P*=.86) | Highly engaging content | Messages fatigue |
| Sabben, G, 2019 | Kenya  Randomized controlled trial  Adolescents (11-14 years) and their parents  30 participants (16 males, 14 females)  Parents: 22 participants (19 females, 3 males) | Gamification app (smartphone)  Features:  Interactive games  Decision support | Acceptability, usability  Engagement (time spent playing the game, response to the game) | Appeal: 90% found the game "very fun," 97% would recommend it to friends, 93% wanted to play more  Engagement: participants played on average 27 hours, 87% played daily, 77% played for more than an hour per session  Usefulness: all participants (100%) indicated that they had learned “a lot” and found the information useful, 93% felt more prepared to handle difficult situations, and 97% felt more confident in saying no when pressured  Parents' perspective: generally positive, with most parents reporting increased interest in topics such as HIV and positive behavioral changes in their children | Engaging content and gamification  Parental support  Easy to use and understand for the majority of adolescents | Content perceived as confusing or uncomfortable |
| Muhindo, R, 2021 | Uganda  Case and control study with pre- and post-intervention assessments  Female sex workers in two cities (intervention and control)  Median age (intervention: 26 years; IQR 21-31; and control: 28 years; IQR 24-32) at baseline, 27 years (236 participants; case 136 and control 100) | Mobile SMS  Features:  SMS reminders on syphilis and HIV testing  Weekly peer education sessions by trained FSW peer educators | Uptake of HIV and syphilis testing | Intervention group:  82% tested for HIV and 81% for syphilis after the intervention (compared to 57% and 35% at baseline, respectively)  Control group:  HIV testing unchanged (52% vs 54%), and syphilis testing decreased (from 39% to 26%)  Peer educators influenced 63.6% of syphilis tests and 48.2% of HIV tests, compared to SMS reminders (57.3% for syphilis, 39.5% for HIV) | Peer support as positive role models influenced HIV and syphilis testing uptake through information and emotional support  Support from local bar maids and hotspot mapping | Low literacy levels among FSW may have limited understanding of SMS messages  Lack of emotional and affiliation support in SMS reminders compared to peer education, reducing their standalone efficacy  In-migration from intervention to control group potentially contaminating control group results |
| Mbotwa, C, 2023 | Tanzania  Pragmatic quasi-experimental trial  Female sex workers (FSW), median age of 26 years  Education: 58.9% had secondary education  Marital status: 75.5% were never married  470 FSW | Smartphone-based mobile application  Features: medication reminders, access to PrEP information, web-based consultations with doctors or peer educators, and anonymous web-based discussions between PrEP users | PrEP retention and app use | About half (52.6%) of study participants were optimal users of the app (ie, they had used at least three app functionalities during the first 30 days after having the app installed on their phones)  Overall retention in PrEP services at 1 month was 27.7%  Retention was significantly higher among optimal app users (37.7%) than sub-optimal users (16.6%) | Availability of web-based consultations and PrEP information through the app | Stigma  Unfriendly legal environment (criminalization of commercial sex) |
| Hovarth, K, 2021 | Uganda  Longitudinal (single-arm pilot) study  Sexually active adults (≥18 years). Average age of participants: 26 years; majority were single (68.4%); students made up 50.5% of total sample, with 74.7% having a tertiary/university or higher education  95 study participants | Telehealth (hotline, mobile SMS, social media)  Features:  Teleconsultations  Health information for behavioral change via SMS  Social media for marketing | Feasibility and acceptability | Feasibility: 94% retention at 3 months; 86% of participants ordered HIV self-testing kits; 69% of kits were used by participants themselves; 31% (n=53) were ordered for others or partners  Acceptability: 94% of participants reported being very satisfied with HIV self-testing; 74% found the test very easy to complete; 93% found the instructions very clear | Convenience of kit delivery and telehealth support  Participants liked the privacy and autonomy provided by the intervention | Older women had slightly lower acceptability ratings.  Limited familiarity with HIV self-testing at baseline (only 13% had used HIV self-testing before) |
| Byonanebye et al, 2021 | Uganda  Randomized controlled trial (RCT) (intervention arm receiving IVR OR SMS and control arm on standard of car)  Adult (≥18 years), both ART-naïve and ART-experienced  600 (413 female, 68.8%) people living with HIV | Interactive voice response (IVR) and mobile SMS | Feasibility, acceptability, and impact of IVR technology on the quality of life (QoL) and viral suppression in people living with HIV in Uganda | *Feasibility:* The study found that 346,286 outbound calls were made, of which 52.8% were answered and 40.7% were uninterrupted until the end of the call  *Acceptability*: 99.3% of participants in the intervention group chose to receive reminders via IVR, and only 0.7% chose SMS text messaging. This suggests that IVR was highly acceptable among participants  *Specific impact*: Among those who used the IVR tool more intensively, there were statistically significant improvements in overall QoL (*P*=.02), mental health domain scores (*P*=.008), and viral suppression (*P*=.006), suggesting that the tool had a positive impact on those who used it regularly. Additionally, these participants also showed better adherence to clinic appointments | Minimal human interaction; therefore, easily scalable and cost-effective  Flexibility in choice on when to receive health tips (ideal time) | Software failure that can interrupt service delivery  Busy schedule of users resulting in not using the services (unable to listen through the entire IVR) |
